# TMS-EEG perturbation biomarkers for Alzheimer’s disease patients classification

**DOI:** 10.1038/s41598-022-22978-4

**Published:** 2023-05-11

**Authors:** Alexandra-Maria Tăuƫan, Elias P. Casula, Maria Concetta Pellicciari, Ilaria Borghi, Michele Maiella, Sonia Bonni, Marilena Minei, Martina Assogna, Annalisa Palmisano, Carmelo Smeralda, Sara M. Romanella, Bogdan Ionescu, Giacomo Koch, Emiliano Santarnecchi

**Affiliations:** 1 Precision Neuroscience and Neuromodulation Program & Network Control Laboratory, Gordon Center for Medical Imaging, Department of Radiology, Massachusetts General Hospital, Harvard Medical School, Boston, MA USA; 2 Berenson-Allen Center for Noninvasive Brain Stimulation, Department of Neurology, Beth Israel Deaconess Medical Center, Harvard Medical School, Boston, MA USA; 3 Department of Neuroscience and Rehabilitation, Section of Human Physiology, University of Ferrara, 44121 Ferrara, Italy; 4 Santa Lucia Foundation, 00179 Rome, Italy; 5 AI Multimedia Lab, Research Center CAMPUS, University Politehnica of Bucharest, 061344 Bucharest, Romania; 6 Siena Brain Investigation & Neuromodulation Lab (Si-BIN Lab), Department of Medicine, Surgery, Neurology and Clinical Neurophysiology Section, University of Siena, Siena, Italy; 7 Department of Education, Psychology and Communication, University of Bari Aldo Moro, Bari, Italy; 8 Department of Psychology, La Sapienza University, Via dei Marsi 78, 00185 Rome, Italy

**Keywords:** Electroencephalography - EEG, Data processing, Machine learning, Alzheimer's disease, Neurodegeneration

## Abstract

The combination of TMS and EEG has the potential to capture relevant features of Alzheimer’s disease (AD) pathophysiology. We used a machine learning framework to explore time-domain features characterizing AD patients compared to age-matched healthy controls (HC). More than 150 time-domain features including some related to local and distributed evoked activity were extracted from TMS-EEG data and fed into a Random Forest (RF) classifier using a leave-one-subject out validation approach. The best classification accuracy, sensitivity, specificity and F1 score were of 92.95%, 96.15%, 87.94% and 92.03% respectively when using a balanced dataset of features computed globally across the brain. The feature importance and statistical analysis revealed that the maximum amplitude of the post-TMS signal, its Hjorth complexity and the amplitude of the TEP calculated in the window 45–80 ms after the TMS-pulse were the most relevant features differentiating AD patients from HC. TMS-EEG metrics can be used as a non-invasive tool to further understand the AD pathophysiology and possibly contribute to patients’ classification as well as longitudinal disease tracking.

## Introduction

Alzheimer’s disease (AD) is a neurodegenerative disorder causing the most common type of dementia worldwide. In the US alone, the prevalence in 2020 was of 11.3% and it is estimated the number of patients living with clinical AD will double by 2060^1^. Despite the high prevalence, an effective treatment for AD is not yet available and research is still being conducted on elucidating AD pathophysiology^2,3^. The latest evidence from both human and animal studies suggests that the causes of the cognitive decline and memory deficits experienced by Alzheimer’s disease patients might be due to brain network abnormalities, including altered neuronal oscillations^4–6^, disruption of long range connections^7^ and default mode network (DMN) nodes^8^, hyperexcitability^2,9,10^ and altered interneurons’ communication^11^. AD neuropathological hallmarks are represented by two different types of aberrant aggregated proteins, namely hyperphosphorylated tau that accumulates intracellularly in the form of neurofibrillary tangles and amyloid-*β* (A*β*) which accumulates extracellularly in the form of senile plaques^2^. From a biological perspective, accumulation of insoluble amyloid products within the synaptic clefts are thought to interfere with both glutamatergic and GABAergic membrane receptor activity determining a shift in neurotransmission excitatory/inhibitory balance towards a condition of hyperexcitability. This has been reported already in the early stage of the disease, even before amyloid plaques accumulation and neurodegeneration occur^12^. Based upon these findings, hyperexcitability can be considered as an AD early pathological alteration resulting in neuronal firing abnormalities, aberrant epileptiform activity and changes in brain oscillatory rhythms as shown in several animal models of AD^9,13^. Memory deficits are hypothesized to be directly caused by a compensatory response to hypersynchrony which interfere with normal neuronal functioning essential in memory processes^2,5^. Hyperexcitability is also considered a characteristic of the AD brain. It has been observed in animal models as well as in human studies^10,11,14,15^, with measures of motor cortex excitability being used for characterizing disease severity^16–20^.

In the last decade, evidence of the primary motor cortex (M1) hyperexcitability in patients affected by AD using non- invasive techniques such as Transcranial Magnetic Stimulation (TMS) has been revealed^17,19–21^ mostly through the use of peripheral motor-evoked potentials. By recording motor evoked potentials (MEP) as a response to applied TMS, cortex excitability measures such as motor threshold at rest (RMT), cortical silent period (CSP), intracortical inhibition (ICI) etc. can be easily obtained. These types of motor cortex excitability measures showed in the recent past that motor cortical excitability is heightened in AD patients, which is highly indicative of a rearrangement of mortor cortical maps^19,22^. Recently, the combination of TMS and electroencephalography (EEG) recordings allow the possibility to study the excitability outside the M1. This is critical since in AD prominent neuropathological abnormalities affect the default mode network, for which the dorso-lateral prefrontal cortex (DLPFC) and the posterior parietal cortex are the main hubs. TMS-EEG allows to stimulate a precise cortical area and, at the same time, to monitor neural activity both in the stimulated area and in the interconnected networks with an excellent temporal resolution in a milisecond timescale. In specific, the analysis of the local and global TMS-evoked EEG potentials (TEPs)^23^ and subsequent responses after perturbation of particular brain regions, might allow us to identify sensitive, diagnostic, perturbation-based biomarkers before clinical symptomatology and irreversible neuronal loss occur^24,25^.

Evidence of early TEP responses being abnormal in AD patients in the central and posterior areas have been previously reported while stimulating the M1 or the left DLPFC (L-DLPFC). Correlations were observed between the P30 latency amplitude and cognitive decline in Alzheimer’s disease^26,27^, while early TEP peaks have been shown to differentiate between AD and HC^28^. Bagattini et al.^26^ found P30 components from the right superior parietal cortex predict poorer cognitive performance while stimulating the L-DLPFC. Most studies investigating AD TMS-EEG focus on the early windows of TEPs (earlier than 100 ms), as a measure of excitability differences when compared to TMS in healthy controls (HC). Therefore, in this study we investigated both early and late (> 100 ms) TEP responses. TEPs obtained over the entire scalp by stimulating the L-DLPFC might have potential in serving as a clinical tool for the differentiation of AD and HC. Machine learning methods have often been used in clinical contexts either for providing a diagnosis or helping in the differential diagnosis of diseases. A multitude of sensor modalities such as medical imaging, resting state EEG or speech have been used in the classification of AD patients^29^. Relevant to the present work, TMS-related metrics for M1 cortical excitability have been extracted by Benussi et al.^30,31^ and used with a Random Forest classifier for the differentiation of MCI from AD and for the differential diagnosis of AD and other neurodegenerative dementias. However, TMS evoked EEG responses obtained via TMS-EEG have not been used for classification purposes. Here, we combine TMS-EEG data with machine learning to extract useful information in identifying and characterizing AD.

Specifically, we investigate the response to stimulation in the L-DLPFC as it is a critical area for memory performance. For instance, in mild cognitive impairment an improvement in memory scores has previously been achieved in multiple studies though stimulation of the L-DLPFC^32–34^. Here we aim to use a data driven approach to: (i) propose a selection of data-driven time domain TEP metrics for identification of AD patients, (ii) identify the most relevant local and distributed metrics of altered brain physiology in AD.

## Methods

### Data collection

#### Participants

AD patients and HC participants were recruited at the Santa Lucia Foundation (Rome, Italy) between January 2014 and June 2020. A total of 126 patients complaining of memory deficits were screened according to the current diagnostic criteria for AD^35^. The study inclusion criteria were as follows: (i) age between 50 to 85 years, (ii) a clinical dementia rating score of 0.5 to 1, (iii) mini-mental state examination (MMSE) score of 18 to 26 at screening, (iv) one caregiver available (v) had undergone treatment with acetylcholinesterase inhibitor for the last 6 months. Exclusion criteria were defined for patients with extrapyramidal signs, history of stroke, other neurodegenerative disorders, psychotic disorders, signs of cerebrovascular disease on MRI and if they had been under treatment six months prior to recruitment with antipsychotics, antiparkinsonian, anticholinergics and antiepileptic drugs.

A total of 38 AD patients and 17 aged-matched controls were included in the analysis based on the recruitment selection criteria and the quality of the recorded data. The TMS-EEG protocol was applied at the time of enrollment. Clinical and neuropsychological data was collected at the time of enrollment. The study was approved by the review board and ethics committee of the Santa Lucia Foundation and was conducted following the principles outlined in the Declaration of Helsinki and the International Conference on Harmonization Good Clinical Practice guidelines. Written informed consent was obtained from all participants or their legal representatives, while being informed that participation is optional and they could withdraw at any time.

#### Transcranial magnetic stimulation-TMS

TMS was carried out using a magnetic biphasic stimulator connected to a figure-of-eight coil with a 70-mm diameter that generates 2.2 T as maximum output (Magstim Company, Whitland, UK). Individual T1-weighted MRI volumes were used as an anatomical reference. To target the L-DLPFC, the coil was positioned over the junction of the middle and anterior thirds of the middle frontal gyrus, corresponding to an area between the center of Brodmann Area (BA) 9 and the border of BA9 and BA46. During L-DLPFC stimulation, the coil orientation was set 45° away from the midline^21,24,36^. Stimulation intensity was based on a distance-adjusted motor threshold considering the individual coil-to-cortex distance^24,37^ (see Supplementary Methods).

This procedure provides a more accurate index of cortical excitability and improves the efficacy of motor threshold calibrated TMS^37^.The intensity of stimulation of single-pulse TMS was set at 90% of the adjusted motor threshold. To ensure a high degree of reproducibility across neurophysiological assessments, the coil position was constantly monitored using the Softaxic neuronavigation system. TMS was delivered in blocks of 120 single-pulses with an inter-stimulus interval of 2–4 s.

#### EEG recordings

During all the TMS-EEG recordings, participants were seated on a comfortable armchair in a soundproof room in front of a computer screen. They were instructed to fixate on a white cross (6 × 6 cm) positioned in the middle of the screen and to keep their arms in a relaxed position. During TMS-EEG, participants wore in-ear plugs which continuously played white noise that reproduced the specific time-varying frequencies of the TMS click in order to mask the click and avoid possible auditory event-related potentials. To further improve auditory masking, participants also wore ear defenders (with a signal-to-noise ratio of 30) on top of the earphones. The intensity of the white noise was adjusted for each individual by increasing the volume (always below 90 dB) until the participant was sure the TMS-induced click sounds could no longer be heard. This is an optimized procedure that we tried in a previous study aimed at isolating peripheral reponses, i.e. auditory and somatosensory potentials, related to TMS^38^. EEG was recorded with a TMS-compatible DC amplifier (BrainAmp, BrainProducts GmbH, Munich, Germany) from 62 TMS-compatible Ag/AgCl pellet electrodes mounted on an elastic cap. Additional electrodes were used as a ground and reference. The ground electrode was positioned in AFz, while the reference was positioned on the tip of the nose. EEG signals were digitized at a sampling rate of 5 kHz. Skin/electrode impedance was maintained below 5 kΩ.

### Data processing and classification overview

**Figure 1:**
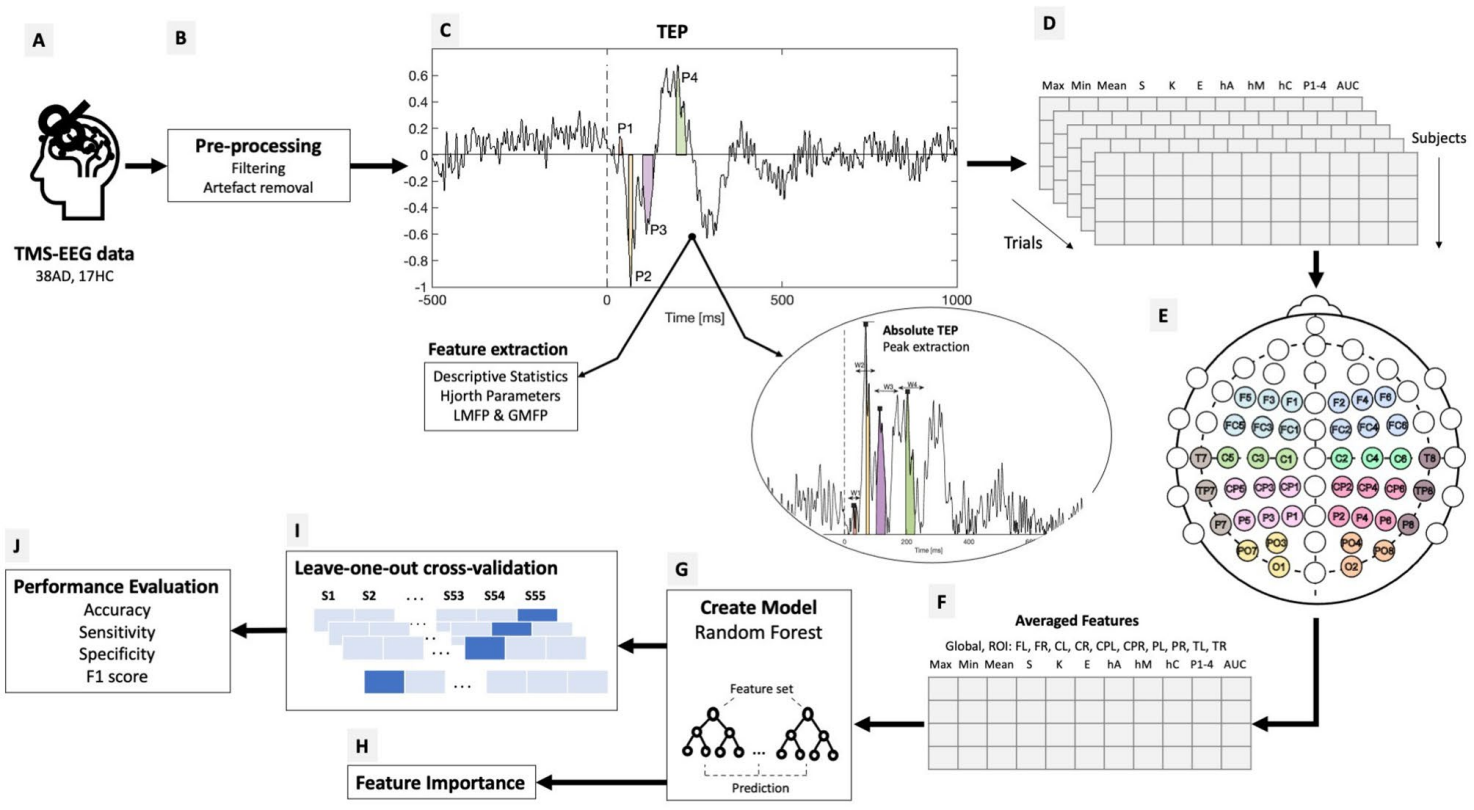
Overview of the TMS-EEG data processing for classifying AD and HC participants; (**A**) TMS-EEG data from both AD and HC participants are taken as input. (**B**) Prior to extracting features, the data is pre-processed to eliminate unwanted interference as well as TMS related artifacts, eye blinks or movement. (**C**) Several features are extracted from the TMS-EEG evoked potentials including descriptive statistics, Hjorth parameters and AUC of LMFP or GMFP, as well as peak values in early windows of the TEP (25–40 ms, 45–80 ms, 85–150 ms and 160–250 ms). (**D**) All features are extracted per individual trial from each recorded channel for each subject. (**E**) Ten regions of interest (ROI) are defined: *FL* frontal left, *FR* frontal right, *CL* central left, *CR* central right, *CPL* central-parietal left, *CPR* central-parietal right, *POL* parietal-occipital left, *POR* parietal-occipital right, *TL* temporal left, *TR* temporal right. The electrodes from each individual ROI are depicted with different colors. (**F**) Feature values are averaged over the entire electrode set (global) and on particular ROIs. (**G**) Different combinations of features are fed into a random forest classifier. (**H**) As a byproduct of classification, an importance value is extracted for each input feature. (**I**) The model is validated in a leave-one-out cross-validation scenario. (**J**) Finally, the metrics used for evaluating the classification performance are accuracy, sensitivity, specificity and F1 score. *Max* maximum amplitude, *Min* minimum amplitude, *S* skew, *K* kurtosis, *E* energy, *hA* Hjorth Complexity, *hM* Hjorth Mobility, *hC* Hjorth Complexity, *P1-4* peaks from defined windows (25–40 ms, 45–80 ms, 85–150 ms, 160–250 ms), *AUC* area under the curve for LMFP and GMFP.

The overall framework for processing TMS-EEG data is presented in Fig. 1. The collected data is pre-processed and divided in trials. Trials are selected based on the timing of the TMS pulse: the baseline period is considered 500 ms to 200 ms prior to the TMS pulse to eliminate filtering artefacts, while the response is analyzed 1000 ms after the pulse. Most TMS-EEG studies analyze evoked potentials in the first 100 ms after stimulation. Here we aim to also investigate later responses present in the EEG after stimulation.

#### Pre-processing

EEG data collected during TMS stimulation is subject to multiple sources of interference and is thus pre-processed to eliminate confounding factors in further analysis^21^. The EEG was divided into two second segments: 1 s prior to the TMS pulse and 1 s after. The signal recorded exactly during the application of the TMS pulse was removed (1 ms prior to the pulse and 10 ms after) and a cubic interpolation was performed. Afterwards, the data was down sampled to 1000 Hz and a zero-phase Butterworth band pass filter was applied between 1 and 80 Hz. Excessively noisy epochs were excluded from the analysis by visual inspection. Muscle activity, eye movements, blinks and residual TMS artifacts were eliminated by performing an independent component analysis (ICA) and applying previously established criteria^21,39,40^. Re-referencing was performed to the overall electrode average. Prior to performing further analysis, the first 500 ms from each epoch were eliminated. For each subject dataset, an average of 8 ± 2*.*3 ICA components were removed. The number of removed epochs was always < 10% of the total number of epochs recorded i.e. not more than 12 epochs were removed. On average, for each subject dataset 10 ± 1*.*8 epochs were removed.

#### Feature extraction

**Figure 2:**
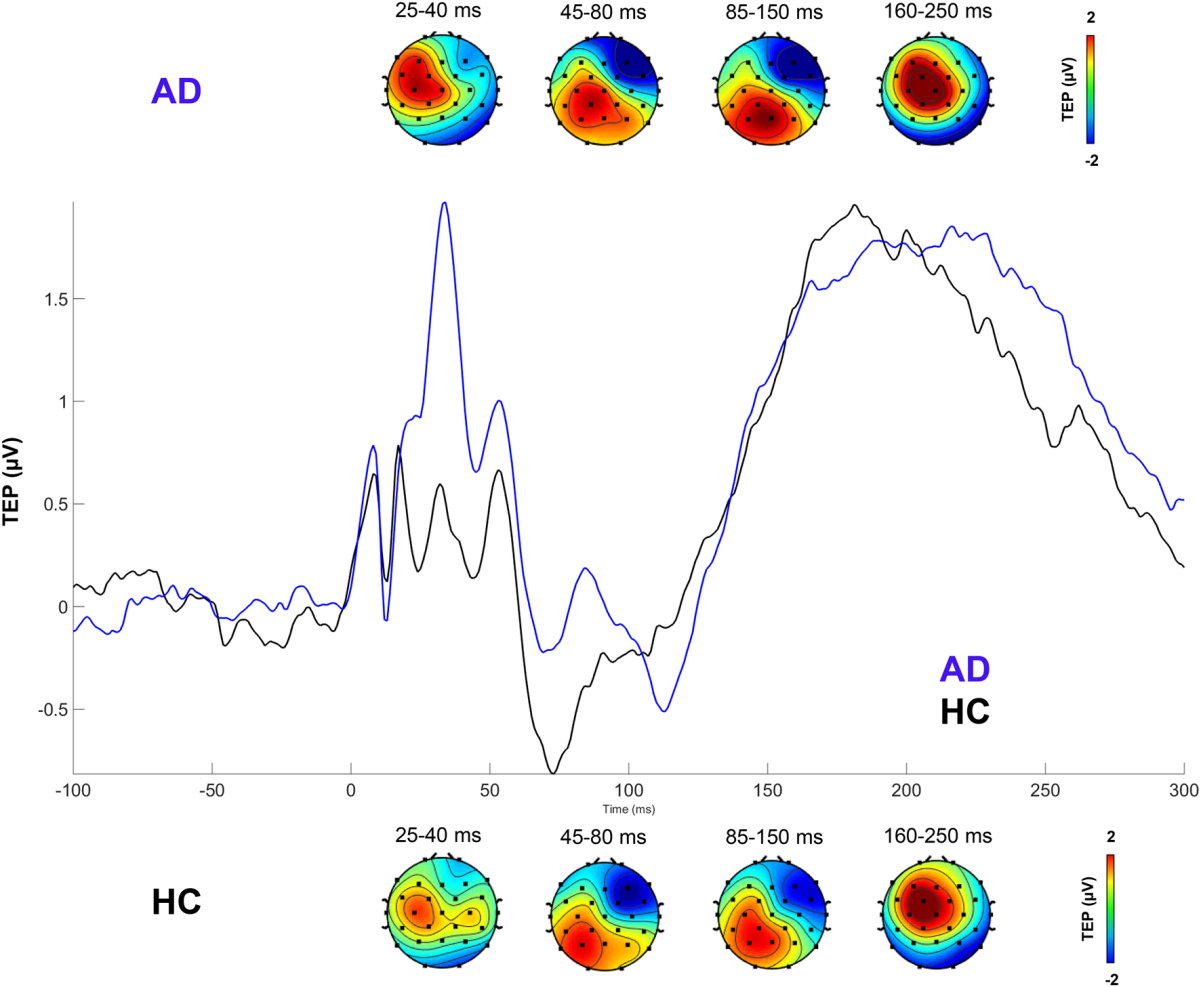
Example of grand averages of TEPs in all AD patients and HC.

The selection of features to be included in the classification algorithm was done based on prior literature that highlights time domain differences in the TEP^41^ between AD and HC subjects, as well as on features typically used in the characterization of time series and more specifically for EEG signal analysis^42^.It is expected these would reveal relevant differences as different characteristics can be observed also in the grand averages of the signals after stimulation in AD patients and HC (see Fig. 2). All features were extracted on individual trials using Matlab R2019a.

*Descriptive statistics* The EEG signal after the TMS pulse was characterized by statistical measures that consider the signal samples as variables. Prior to extracting statistical measures, the EEG signal was normalized between -1 and 1. The measures used included the maximum, minimum, mean, skew and kurtosis. The mean of a signal represents the first statistical moment and characterizes the overall amplitude present in the analyzed window. The skew is the third statistical moment and provides insights into the symmetry of the samples’ distribution. The kurtosis is the fourth statistical moment and indicates how tailed the samples’ distribution is.

*Hjorth parameters* The Hjorth activity, mobility and complexity of an EEG signal are statistical parameters based on the variation of the signal in time domain and can provide a parallel to frequency content characterization without applying additional transforms^43^. They are a fast tool for the characterization of time series variations and are effective in various EEG applications, including AD detection^42,44^. Activity was calculated as the variance of the signal after the TMS pulse. Mobility is representative for the standard deviation of the power spectrum, while the complexity is representative of the similarity of the signal to a sine wave function.

*Signal Energy* Changes in the EEG signal due to the TMS perturbation can also be characterized through the energy content of the signal. Signal energy is calculated as the mean area under the squared magnitude of the EEG signal after the TMS pulse.

*TEP peaks* The latencies and peak amplitude of TMS evoked potentials have been used to characterize the response to stimulation in various recording conditions or the reaction to TMS pulses in different pathologies^19,27,28,45^. As the TEPs show significant inter-individual differences^46^ and the amplitude and latency difference in TEPs from AD and HC are still a topic of research, peaks were detected in four specific time windows after the TMS pulse: 25–40 ms (P1), 45–80 ms (P2), 85–150 ms (P3), 160–250 ms (P4). The maximum value was selected from each window based on the absolute value of the signal. To avoid detecting local maxima, a window around the maximum value was taken and the peak amplitude was considered as the mean absolute signal amplitude in this window^47^. Before 80 ms, the mean value was computed between 5 ms before and after the maximum value. After 80 ms, the peak was the mean value between 15 ms before and after the detected maximum.

*Mean Field Power* The mean field power (MFP) was computed both globally and for different ROIs^48^. For the global mean field power (GMFP), the field potential is computed over all the channels available for that patient. The local mean field power (LMFP) is calculated only for electrodes in the defined ROIs. The area under the curve (AUC) is extracted from the GMFP and LMFP and it is taken as input for classification.

#### Averaging

All features were extracted from individual trials from each channel (except LMFP and GMFP). These were averaged per individual channel and afterwards averaged on the global electrode set or for the various ROIs defined for each subject. Ten regions of interest are defined: FL-frontal left (F1, F3, F5, FC1, FC3, FC5), FR-frontal right (F2, F4, F6, FC2, FC4, FC6), CL-central left (C1, C3, C5), CR-central right (C2, C4, C6), CPL-central parietal left (CP1, CP3, CP5, P1, P3, P5), CPR-central parietal right (CP2, CP4, CP6, P2, P4, P6), POL-parietal occipital left (PO3, PO7, O1), POR-parietal occipital right (PO4, PO8, O2), TL-temporal left (T7, TP7, P7), TR-temporal right (T8, CP8, P8). These are illustrated in Fig. 1E.

#### Classification and evaluation

The selected algorithm for creating a model differentiating between AD and HC was a Random Forest (RF) classifier. The RF classifier is an ensemble machine learning technique that combines the output from several decision trees. The final solution is based on majority voting^49,50^. RF has previously shown good results in clinical classification problems^31,51^. A total of 100 trees were used in the random forest with a minimum number of one sample per leaf. To account for the variability in the result, the algorithm was run 100 times for each experiment. These numbers were set after several preliminary optimization experiments.The classification analysis and corresponding evaluation was conducted in Python v3.7.

The RF classifier also has the advantage of having inherent feature selection based on gini impurity^52^ which allows to assign a feature importance. As a by-product of the RF classifier training, the feature importance value can provide an indication of how relevant a feature is in distinguishing between AD and HC. The presented results are a sum of the feature importance values for each cross-validation split and run of the algorithm. Based on the importance ranking, some of the features were further analyzed with independent t-tests with Bonferroni correction for AD and HC conditions.

Several combinations of time features were used for classification to test the discriminative power of different feature combinations from the scalp. The following configurations were experimented with: (i) Features averaged globally over the entire available electrode set, (ii) ROI averaged-features were averaged for all individually defined ROIs and added together as input to investigate the impact of reducing the spatial input information; (iii) Individual ROI based-features were averaged per ROI and a classification model was created for each individual ROI feature set to study the topographic impact on classification performance.

The TMS-EEG dataset contains an unbalanced number of classes (AD and HC). This inequality biases the classifier towards the predominant class as there are more instances to learn from, resulting in poorer performance in discriminating the minority class. To counter this effect, we make use of the Synthetic Minority Over-sampling Technique (SMOTE)^53^. Synthetic features are created in the feature space of the minority class by selecting *k* neighbors of the current sample and adding an intermediate synthetic feature value. Results are presented both with and without SMOTE applied to the training feature set.

The performance of the classification of AD patients is evaluated with a leave-one-subject-out cross-validation method. The training set consists of *k − *1 subjects used as input for creating the model, while the *k*^*th*^ subject is used as a test for calculating the performance metrics. The process is repeated until all subjects have been tested. The reported performance is the average over all 100 runs of the leave-one-subject-out procedure. The metrics used for performance evaluation are accuracy, sensitivity, specificity and *F*_1_ score^54^.

## Results

### Subjects

A total of 38 AD patients and 17 HC were included in the present study. A summary of the demographics of the patients included in our study is available in Table 1. Results of the neuropsychological assessment are also presented.

**Table 1:** Subject demographics and clinical characteristics at the moment of TMS-EEG data collection.

T0-initial assessment	AD participants	HC participants
Total Number	38	17
Sex (female-number, percentage)	23 (60.53%)	10 (58.82%)
Age (M ± SD)	72.63 ± 6.28	71.11 ± 6.77
Education (M ± SD)	12.60 ± 11.41	11.11 ± 4.66
MMSE score (M ± SD)	18.94 ± 6.07	29.06 ± 1.34
CDR-SB score (M ± SD)	4.53 ± 2.50	–
ADAS-Cog score (M ± SD)	24.67 ± 9.70	–
ADCS-ADL score (M ± SD)	57.44 ± 12.49	–
NPI score (M ± SD)	10.42 ± 8.62	–
FAB score (M ± SD)	10.22 ± 3.83	–

*MMSE* mini mental state examination, *CDR-SB* clinical dementia rating scale sum of boxes, *ADAS-COG* The Alzheimer’s disease assessment scale-cognitive subscale, *ADCS-ADL* Alzheimer’s disease cooperative study-activities of daily living, *NPI* neuropsychiatric inventory, *FAB* frontal assessment battery.

### Classification performance

Multiple classifications were performed for identifying AD from HC patients based on combinations of the time domain features averaged globally, over the ROIs set or a combination of both. Results are summarized in Table 2. Higher values for accuracy, sensitivity and *F*_1_ score were obtained when using a balanced feature set obtained with the SMOTE technique as input. A significant difference can be seen in the obtained sensitivity when using the unbalanced and balanced feature set. Applying the balancing technique doubles the sensitivity value.

**Table 2:** Classification results obtained while using time domain features both with the balanced (SMOTE) and unbalanced feature set.

Experiment	Accuracy	Sensitivity	Specificity	*F*_1_ score
Unbalanced dataset				
Global	83.1	70.05	88.94	79.88
ROI	73.34	45.64	85.73	66.35
Global & ROI	77.8	54.82	88.07	72.28
Balanced dataset				
Global	92.05	96.15	87.94	92.03
ROI	85.26	92.73	77.78	85.14
Global & ROI	87.14	91.55	82.73	87.10

**Figure 3:**
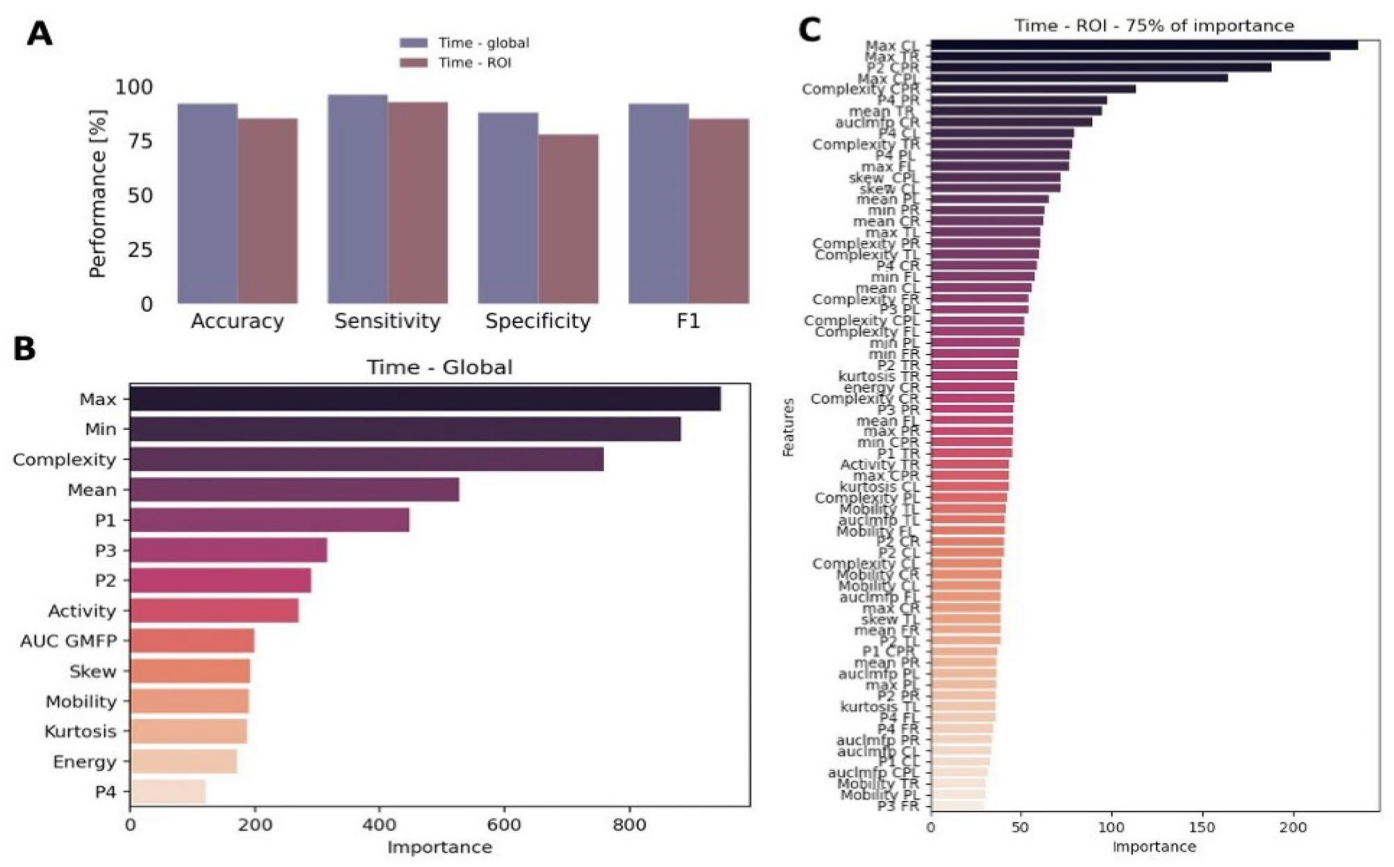
Classification performance and feature importance. (**A**) Overall view of classification performance on global and ROI based balanced feature set. (**B**) Global feature ranking based on RF importance. (**C**) ROI feature ranking based on RF importance.

The highest classification performance of 92.05%, 96.15% and 87.94% accuracy, sensitivity and specificity respectively, was obtained using only global values as input for classification. When using all features averaged on ROIs as input, accuracy, sensitivity and specificity dropped by approximately 7%, 4% and 10% respectively. A comparison of global and ROI based classification performance is available in Fig. 3A. When combining information from both global and ROI based features, the performance does not increase but remains between the performance obtained with the two feature sets independently. All classifications were performed with a Random Forest algorithm. Other classifiers were also tested. The comparison of the results obtained from a Decision Tree and k-Nearest Neighbors classifiers is available in Supplementary Fig. 1. The variation of some of the features with respect to baseline was also used as input for classification. The performance in this case remains in the same range with no substantial variations. The results are available in Supplementary Fig. 2.

For comparison purposes, the RMT values recorded for both AD and HC were used directly as input for the RF classifier. The performance in terms of accuracy, sensitivity, specificity and F1 score was of 61.4%, 64.63%, 58.18% and 61.36% respectively.

**Figure 4:**
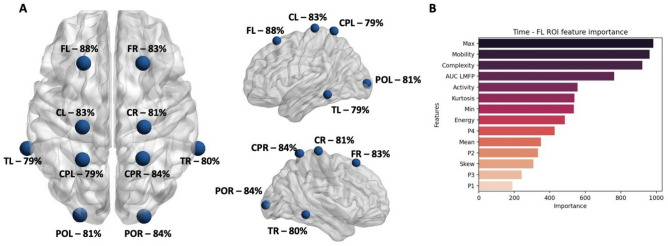
(**A**) Accuracy of the classification using features from individual ROIs as input. The graphical location of the ROIs is representative to the position of the specific electrodes included^74^. (**B**) Ranking of features based on feature importance for the FL ROI. *FL* frontal left, *FR* frontal right, *CL* central left, *CR* central right, *CPL* central-parietal left, *CPR* central-parietal right, *POL* parietal-occipital left, *POR* parietal-occipital right, *TL* temporal left, *TR* temporal right.

Additionally, to investigate the potential for AD identification from TEP responses from individual brain regions, classifications were performed on features from individual ROIs as input. A summary visualizing the accuracies obtained from each ROI is available in Fig. 4A using the balanced versions of features for individual ROIs. All accuracy values were approximately in the same range, with variations of less than 10%. A slightly higher accuracy of 88% was obtained for FL, while the lowest of 79% on CPL and TL. For FR and CL the accuracy was equal to 83%, for CR and POL it was 81%, while for CPR and POR it was 84%. A similar trend in sensitivity, specificity and F1 score was observed when using individual ROIs as the ones reported for the global and all ROIs based performance. The feature importance analysis results in a similar ranking of features as in the case of using all ROIs for classification, with maximum amplitude values and Hjorth parameters having the highest overall importance.

### Feature analysis

Feature importance was extracted when training RF on global and ROI based feature sets. The feature ranking is displayed in Fig. 3B for the global RF classification, while Fig. 3C shows the feature ranking for the ROI based classification. For the ROI based feature ranking, only the first 75% of features in terms of importance value were displayed as there are a total of 150 metrics. In both cases, the maximum amplitude is leading in terms of classification importance. A different feature order follows in case of global and ROI based importance. For the global feature set, the greatest weight comes from the maximum and minimum amplitude values, followed by the computed complexity. In case of the ROI based feature set, the maximum value in the CL and TR regions of interest have the highest weight followed by the P2 peak from the CPR region. A combination of various amplitude based and complexity measures follow in terms of importance ranking. The majority of the features in the top 75% in terms of importance are linked to the complexity, maximum and mean amplitude of TEPs in different ROIs. Figure 4B shows the feature importance ranking for the FL ROI that showed the highest classification accuracy. Similarly to both the global and ROI based approaches, the maximum amplitude ranks highest followed by the Hjorth parameters mobility and complexity.

**Figure 5:**
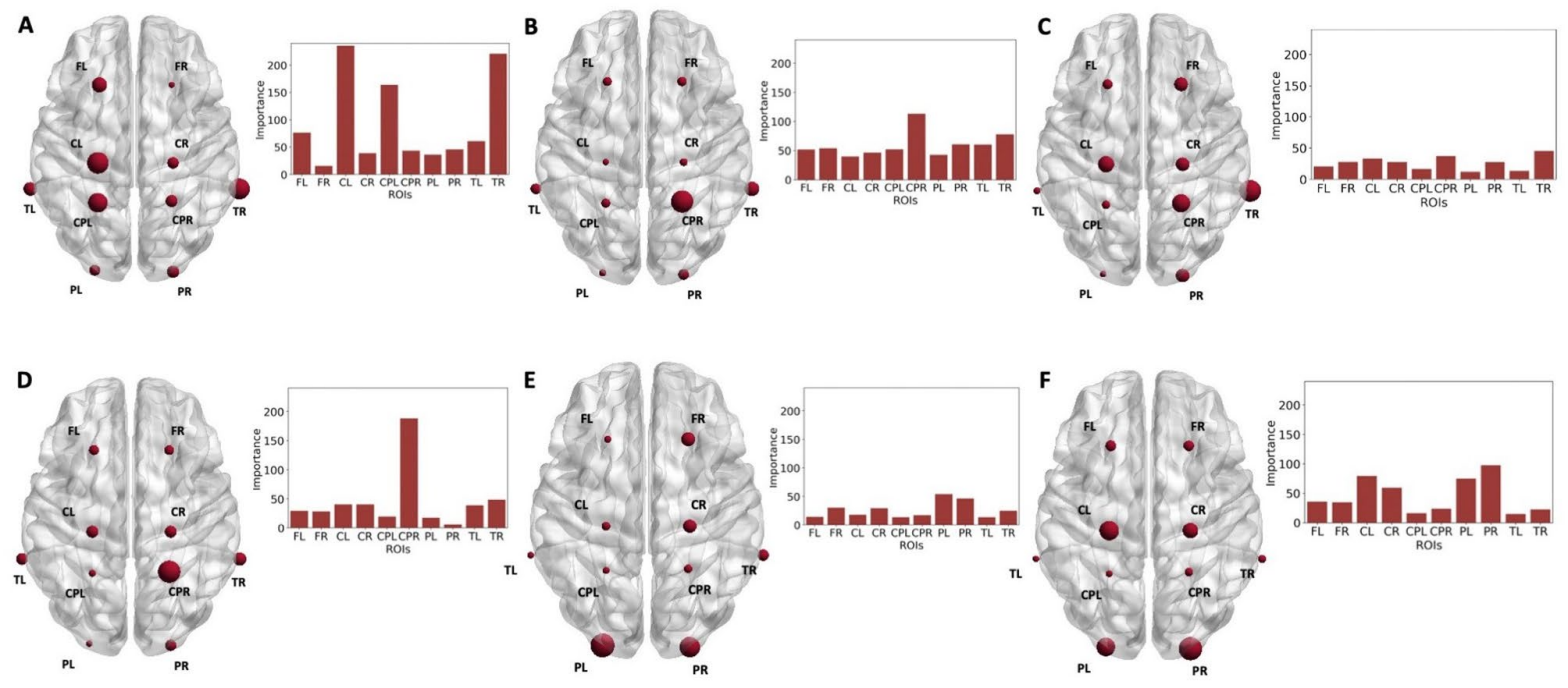
Importance of several features displayed per ROI when identifying AD from HC using RF. (**A**) Maximum value (**B**) Complexity (**C**) Peak P1 (**D**) Peak P2 (**E**) Peak P3 (**F**) Peak P4. *FL* frontal left, *FR* frontal right, *CL* central left, *CR* central right, *CPL* central-parietal left, *CPR* central-parietal right, *POL* parietal-occipital left, *POR* parietal-occipital right, *TL* temporal left, *TR* temporal right.

To better understand the impact of features extracted from particular brain regions in the overall identification of AD patients from HC, a selection of the importance values of some of the features is displayed separately in Fig. 5 for each ROI included in the analysis. Overall, the maximum amplitude of the signal has the heaviest weight in classification, followed by the complexity measure and certain peak values from the CPR, POL and POR regions. The maximum amplitude of the TMS-evoked EEG response from the CL, CPL and TR regions show the highest relevance in differentiating AD and HC. The CPR and TR regions have the highest ranking in terms of complexity and early TEP peaks, P1 and P2. The parietal-occipital regions, POR and POL, have a heavier weight for the later TEP peaks P3 and P4.

**Figure 6:**
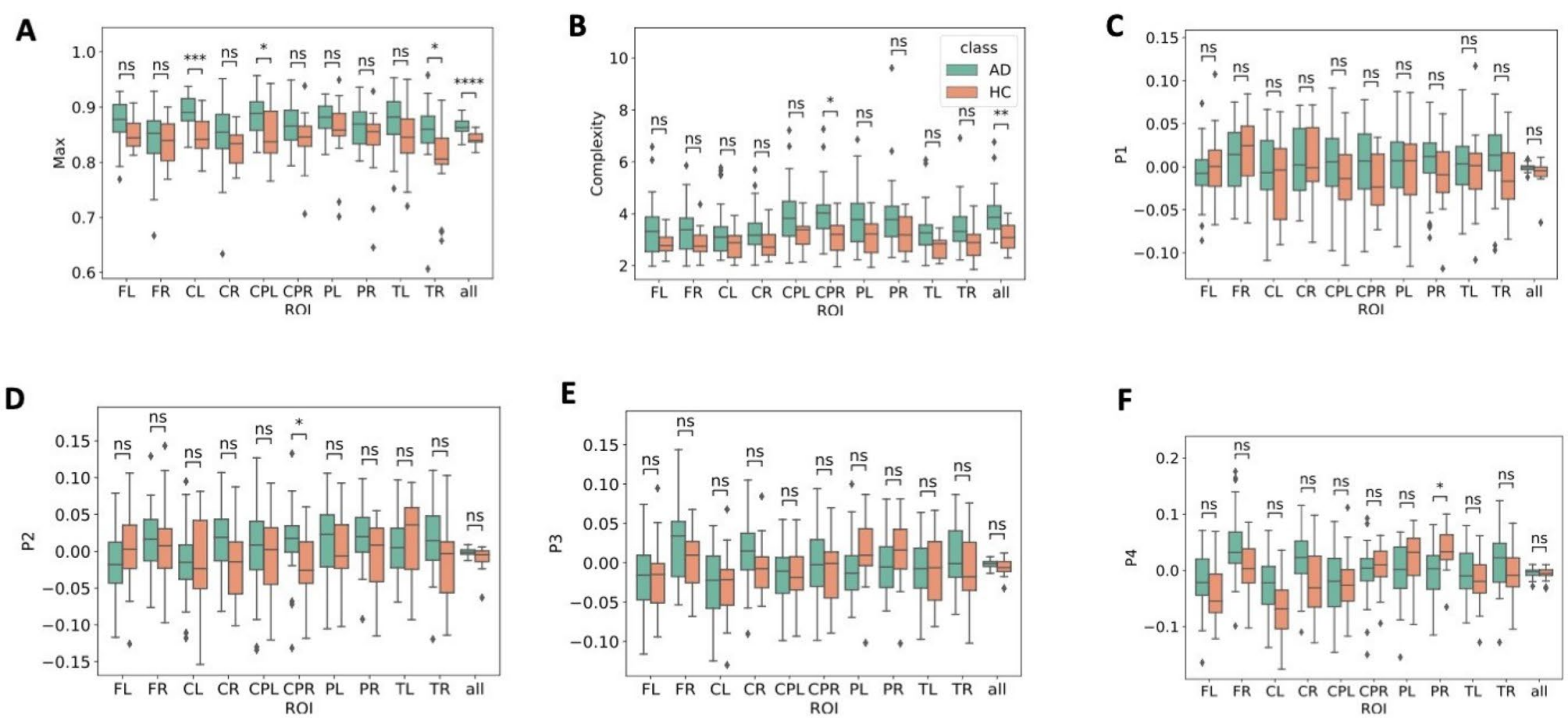
Differences in several feature values for the AD and HC participants. Independent t-tests were carried out: ****P ≤ 0.0001; ***0.0001 < *P* ≤ 0.001; **0.001 < *P* ≤ 0.01; * 0.01 < *P* ≤ 0.05; ns 0.05 < *P* < 1. (**A**) Maximum value (**B**) Complexity (**C**) Peak P1 (**D**) Peak P2 (**E**) Peak P3 (**F**) Peak P4. *FL* frontal left, *FR* frontal right, *CL* central left, *CR* central right, *CPL* central-parietal left, *CPR* central-parietal right, *POL* parietal-occipital left, *POR* parietal-occipital right, *TL* temporal left, *TR* temporal right.

The differences between AD and HC in the maximum amplitude, complexity and peaks (P1, P2, P3, P4) are displayed in Fig. 6. The maximum value of the TMS evoked EEG response in AD patients is generally higher than for HC. Significant differences are reported for the global maximum amplitude (AD: 0.86 ± 0.013, HC: 0.84 ± 0.011, t(55) = 5.52, *P* = 0.00001), for the CL (AD: 0.89 ± 0.029, HC: 0.84 ± 0.037, t(55) = 4.62, *P* = 0.0002), CPL (AD: 0.88 ± 0.034, HC: 0.84 ± 0.047, t(55) = 3.29, *P* = 0.02) and TR (AD: 0.85 ± 0.051, HC: 0.80 ± 0.070, t(55) = 3.10, *P* = 0.03). This is aligned with the feature importance calculated from the RF classification: for the global dynamics classification the maximum amplitude has the heaviest weight, while for the ROI based classification the maximum amplitude in the CL ROI is the most important, followed by the maximum in the TR and CPL regions. All other differences in maximum amplitudes per ROI were non-significant (*P* > 0.05).

Complexity values were significantly different in the globally averaged values (AD: 4 ± 0.79, HC: 3.14 ± 0.51, t(55) = 5.52, *P* = 0.002) and the CPR region (AD: 4.08 ± 1.00, HC: 3.18 ± 0.68, t(55) = 3.28, *P* = 0.02). No significant differences were detected in the P1 peak values for any of the ROIs. For the P2 peak, the CPR region showed a significant difference between AD and HC values (AD: 0.016 ± 0.04, HC: − 0.026 ± 0.04, t(55) = 3.28, *P* = 0.02).No significant difference is reported for the P3 peak. For P4, a significant difference between AD and HC was observed for the PR region (AD: − 0.004 ± 0.04, HC: 0.037 ± 0.03, t(55) = − 3.09, *P* = 0.03).

## Discussion

In this study we have used TMS-EEG measures in the temporal domain to differentiate between AD and HC participants by using a Random Forest classifier. The aim was to investigate the possibility of using different features extracted from the time course of the TMS-evoked response as clinical perturbation biomarkers for AD. The results in terms of classification weight and significant differences in values for the selected amplitude features indicate these could potentially be used as biomarkers of disease. Here we comment on the classification performance and on the features that showed the highest weight in the differentiation of the two conditions.

### Classification accuracy

Our results showed that global features provided a better classification accuracy than using ROI extracted information. AD has been frequently characterized as a disconnection syndrome^55^ where long range connections between hemispheres^56^ and especially between temporoparietal lobes^57^ are lost and there is an increase in local connection density and hyperexcitability^58^. Subsequently, while local changes in specific brain regions can be indicative of AD, our results show that the full impact of the disease might be better assessed while analyzing global brain dynamics. The better classification performance obtained with globally averaged features reflects the impact of the disease on the entire brain. Analyzing global brain dynamics could be more sensitive to pathological brain changes rather than focusing the analysis on specific brain regions only.

The classification accuracy using TMS-EEG time domain information both globally and ROI based was significantly higher than using corticospinal excitability (i.e. RMT) values as input. Although stimulating the motor cortex has previously proven to provide valuable information for brain excitability^59^, the collected RMT values for this dataset do not show a high discriminative power, further supporting the adoption of TMS-EEG measures to understand AD physiology.

Classification accuracy when taking only individual ROI features as input ranged between 79% and 88% on the balanced set. Despite the slight performance drop, the results are still satisfactory to differentiate between AD and HC based on previous attempts^51^. Higher accuracies have been obtained for the ROIs from the right central, right parietal and right frontal lobes. These regions are also indicative for the significant differences in feature values between AD and HC. Tzimourta et al.^60^ report a similar trend in ROI based classification accuracies for AD, where the posterior and central regions outperform the frontal and temporal ROIs. In contrast to other resting state EEG findings^61,62^, we found a higher classification accuracy for ROIs on the right hemisphere. Evidence regarding a right hemispheric dominance in MCI and AD patients compared to HC subjects have been previously reported^63^. No final conclusions regarding AD right versus left hemispheric predominance can be drawn as the differences in performance are also relatively low for the individual regions. Further studies are needed to better clarify this issue.

The highest accuracy when classifying only with individual ROIs was obtained for the FL region. In classification tasks, the result is obtained based on the combination of features used as input. Adding or removing features can increase or decrease the final evaluated performance based on their ability to represent the classes to be differentiated. When using all ROI features as input, the combination results in a different performance and a different feature ranking. The feature ranking presented is based on the RF inherent feature selection. Other types of methods such as feed-forward or backward feature selection that focus on the end classification performance might yield different results and thus a reduced number of features could result in a higher classification accuracy. As an additional check for the relevance of the proposed features in the problem of AD identification, statistical tests between AD and HC were performed which confirmed the relevance of features with the highest weight in classification, for instance the maximum amplitude and complexity of the signal.

### Biomarker identification

In this study, we made use of the feature importance output of the RF classifier to identify the most important measures in the classification of AD patients. The highest-ranking features also show significant differences in their values for AD and HC as revealed by the t-test statistics. For instance, the maximum TEP amplitude was the most relevant feature when classifying based on globally averaged values, whereas for the ROI based classification the maximum amplitude in the CL, TR and CPL regions were in the first five highest ranked features.

Preclinical evidence both from several mouse models of AD and TMS studies on M1 excitability in AD patients showed that a heightened excitability could represent a distinctive and early feature of the disease^59,64^. Further expanding these findings, our results showed that TEPs normalized global maximum amplitude registered after magnetic stimulation of a non-motor area was significantly higher in AD as compared to HC. This could be an indication of an overall stronger response due to the heightened excitability in the AD brain. Furthermore, the amplitude from the CL region located over the left motor cortex brings the strongest weight in the classification problem using ROI values as input, while also showing a significant difference within the two conditions. This could also be linked to the hyperexcitability of the motor cortex as indicated by previous studies^19^, although other investigations showed contradictory results^65,66^. However, since our study did not include TEPs registration following the stimulation of primary motor cortex, conclusions regarding perturbation of which cortical site induce the higher hyperexcitability response in terms of TEPs maximum amplitude cannot be drawn. Moreover, our study only included mild-to-moderate AD patients and the applicability of our findings to the prodromal stages of the disease cannot be assessed.

Early TEP peaks have been used in previous studies to understand cortical excitability by stimulating the motor cortex^19,27,28^ through TMS. In our experiments, no significant differences were detected between AD and HC for P1. However, in the global feature ranking the P1 peak is on the fourth place in terms of weight. When looking at feature importance values, the highest weights are obtained in the right hemisphere on the CPR and TR regions. The findings are aligned with the results of Bagattini et. al^26^. The P60 peak has previously been shown to be indicative of AD hyperexcitability over the FC1 and PC3 electrodes by Ferreri et. al^19^. Here we report significant differences over the CPR region for P2, which includes the time window for the P60 peak. Feature importance values are highest in the CPR region as well, followed by the TR ROI. The CPR ROI is located on top of the right precuneus which is known to be affected in AD. The higher classification performance, as well as the importance of the features from the CPR ROI are indicative of its relevance for AD identification. This is in line with recent evidence showing that repetitive TMS stimulation directly targeting the precuneus is effective in enhancing memory in prodromal AD^24^. In this context, CPR’s P2 peak amplitude variations could be used to monitor cortical excitability changes in AD patients after TMS treatment over precuneus.

The late P3 and P4 peaks of the TEP present a higher weight in the classification in the left and right posterior regions. The P3 peak shows no significant differences, while P4 is significantly different in the POR region. As the perturbation propagates through the different brain regions, differences in the later TEP peaks are expected between AD and HC in the POR and POL regions which could be indicative of dysfunctions in long range connections. Changes in the posterior EEG of Alzheimer’s patients have been previously reported in literature mostly with respect to related frequency changes^4^. Similarly, alterations in maximum amplitude in these regions can be a relevant indication of the presence of AD.

Hjorth complexity of the TMS evoked potential is another feature that showed a high weight in classification both globally as well as for specific ROIs. Similarly to the peak features extracted, the complexity from the CPR and TR regions showed a higher weight in the classification while significant differences are observed for the CPR regions and for the global value.

The use of EEG-based biomarkers shows many advantages from the clinical perspective. As compared to functional and structural neuroimaging techniques (e.g., MRI, PET), EEG allows the detection of static and dynamic brain changes with high temporal resolution, and at a lower cost. Moreover, its combination with brain stimulation techniques such as TMS allows the investigation of the brain’s dynamic response to external perturbations and further improves the detection of subtle pathological features^67^. The use of TMS-EEG in the diagnosis of AD can be an alternative to neuroimaging methods as it can provide clinicians functional and pathophysiological information helpful in distinguishing AD from HC as well as from other neurodegenerative disorders.

### Limitations and future work

The number of AD patients available in the data set used is more than double that of HC. This influences the classification performance as classification algorithms are more prone to recognize the majority class from the dataset used in training. An oversampling technique was used to balance the data; however, this approach might not be representative for real world scenarios. More accurate and representative results would be obtained for the classification if more age-matched healthy controls would be analyzed.

Some of the features extracted showed potential as clinical perturbation biomarkers. However, TMS-EEG data needs to be significantly pre-processed prior to analysis. In addition, TMS can result in non-specific effects, such as auditory and somatosensory stimulation that can affect the EEG response^38^. We adopted several methodological precautions to avoid these artifacts. For instance, to reduce the auditory artifacts response, we used an ad-hoc masking noise^68^. Moreover, to reduce bone conduction of the TMS click and scalp sensation caused by coil vibration, we placed a 0.5 cm foam layer underneath the coil.

The identification of relevant features was based on the inherent feature ranking of the RF classifier. Different types of feature selection methods can be used and might lead to different results. To further strengthen the ranking and selection of relevant features, different methods should be applied, or the stability of the feature ranking should be further evaluated^69^.

Studies on resting state EEG data show a difference in the frequency content of AD patients as compared to HC^70^. In our study, Hjorth complexity also showed a promising possibility as a biomarker of AD. Assessing frequency content of TMS-EEG responses could reveal important features of individual response to perturbation in the healthy and diseased brain. Connectivity patterns are reported to be altered in patients with AD while analyzing resting state EEG^71,72^. Connectivity as revealed after TMS can indicate significant differences between AD and HC, offering the opportunity to look at both functional and directional (effective) connectivity metrics. Combining EEG and brain stimulation has the potential to outperform resting-state EEG alone in characterizing individual cognitive profiles^73^ and possibly constitute markers of neurodegeneration. Future work could focus also on distinguishing between different types of dementia such as Fronto-Temporal dementia or Lewy Body dementia, as well as in characterizing early stages of dementia including MCI and preclinical AD.

## Supplementary Information

Supplementary Information.

## Data Availability

All the data of the present work will be made available upon reasonable request to the corresponding author.
