# Supplementary material for: TMS-EEG perturbation biomarkers for Alzheimer’s disease patients classification
Source: Sci Rep. 2023 May 11;13:7667. doi: 10.1038/s41598-022-22978-4 (PMC10175269; doi:10.1038/s41598-022-22978-4)
Supplement: Supplementary file 1 — Supplementary Information. [file 41598_2022_22978_MOESM1_ESM.docx]

# TMS-EEG perturbation biomarkers for Alzheimer’s Disease classification

Alexandra-Maria Tautan^1,2,5^, Elias P. Casula^4^, Maria Concetta Pellicciari^4^, Ilaria Borghi^4^, Michele Maiella^4^ , Sonia Bonni^4^, Marilena Minei^4^, Martina Assogna^4^, Carmelo Smeralda ^6^, Sara M. Romanella ^1,2,6^, Annalisa Palmisano ^1,2,7^, Bogdan Ionescu^5^ , Giacomo Koch^3,4^, Emiliano Santarnecchi ^1,2^

^1^ Precision Neuromodulation Program & Network Control Laboratory, Gordon Center for Medical Imaging, Department of Radiology, Massachusetts General Hospital, Harvard Medical School, Boston, MA, USA

^2^ Berenson-Allen Center for Noninvasive Brain Stimulation, Department of Neurology, Beth Israel Deaconess Medical Center, Harvard Medical School, Boston, MA, USA;

^3^ Department of Neuroscience and Rehabilitation, Section of Human Physiology, University of Ferrera, Ferrara, Italy

^4^ Santa Lucia Foundation, Rome, 00179, Italy

^5^ University Politehnica of Bucharest, AI Multimedia Lab, Research Center CAMPUS, Bucharest, 061344, Romania

^6^ Siena Brain Investigation & Neuromodulation Lab (Si-BIN Lab), Department of Medicine, Surgery, Neurology and Clinical Neurophysiology Section, University of Siena, Siena, Italy

^7^ Department of Education, Psychology, and Communication, University of Bari Aldo Moro, Bari, Italy

# Supplementary Methods

The distance-adjusted motor threshold was used as a basis for determining the stimulation intensity computed as:

| $adjMT=MT+m*(DSite_{x}-DM1)$ | (1) |
| --- | --- |

where $adjMT$ is the adjusted $MT$ in % of stimulator output, $MT$ is the unadjusted $MT$ in % of stimulator output, $DM1$ is the distance between the scalp and M1 hotspot, $DSite_{x}$ is the distance between the scalp and an x cortical region and m is the distance-effect gradient.

# Supplementary Results

## 1 Comparison with multiple classifiers

Experiments with multiple classifiers were performed on the global and ROI determined feature. Besides the random forest algorithm, models were created with a decision tree (DT) and a k-Nearest Neighbors (kNN) classifier. A comparison of performance is presented in Supplementary Figure 1. Overall, the RF algorithm shows the highest performance in terms of accuracy, specificity and F1 score. In terms of sensitivity, there is a small increase of approximately 3% on the ROI based classification when DT and kNN are used.


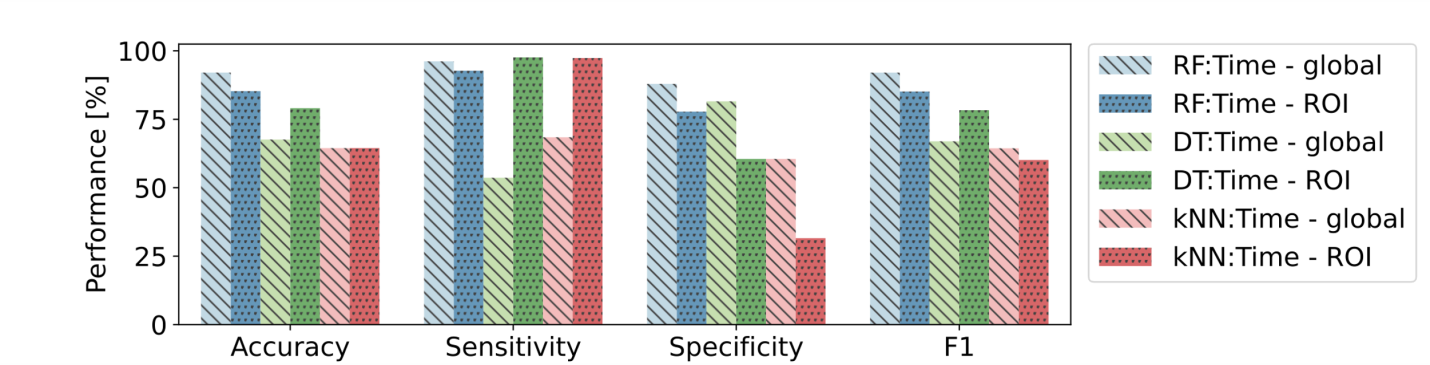


**Supplementary Figure 1:** Comparison of classification performance on the global and ROI based feature set using Random Forest (RF), Decision Tree (DT) and k-Nearest Neighbour (kNN) classifiers. Presented results are on the SMOTE balanced feature set.

## 2 Normalizing feature values to baseline

For some features, the relative change after the TMS pulse with respect to the period after can be revealing in terms of characterizing the TMS perturbation. The rate of change of the values can be calculated and can reveal important information on the response of the subject to TMS. Additional experiments were performed computing the rate of change of some of the feature values with respect to baseline. The same features were extracted both from baseline (500ms to 200ms before the TMS pulse) and after the pulse (up to 1000ms after the TMS pulse). The value obtained after the pulse was divided with the feature value obtained before the pulse, therefore normalizing with respect to baseline. These new normalized features were also used to compute a new model.

Not all features could be normalized as some have values only in terms of response characterization e.g. extracting peaks in different time windows. A summary of the features used in the classification and whether they could be referenced to baseline is available in Supplementary Table 1.

| Feature Name | Description | Normalized to baseline? |
| --- | --- | --- |
| Descriptive Statistics | |  |
| Max | Maximum value of the EEG amplitude after the TMS pulse | Yes |
| Min | Minimum value of the EEG amplitude after the TMS pulse | Yes |
| Mean | Mean value of the EEG amplitude after the TMS pulse. | Yes |
| Skew | Skewness of the EEG signal after the TMS pulse. | Yes |
| Kurtosis | Kurtosis of the EEG signal after the TMS pulse. | Yes |
| Hjorth Parameters | |  |
| Hjorth Activity | Variance of the EEG signal after the TMS pulse. | Yes |
| Hjorth Mobility | Square root of the ratio between the first derivative of the variance of the signal and the variance of the signal. | Yes |
| Hjorth Complexity | Ratio between the mobility of the first derivative of the signal and the mobility of the signal. | Yes |
| Signal Energy | |  |
| Energy | Sum of square root of samples | Yes |
| TEP peaks | |  |
| P1 | Maximum value determined 25 to 40ms after the TMS pulse | No |
| P2 | Maximum value determined 45 to 80ms after the TMS pulse | No |
| P3 | Maximum value determined 85 to 150ms after the TMS pulse | No |
| P4 | Maximum value determined 160 to 250ms after the TMS pulse | No |
| Mean Field Power | |  |
| AUC LMFP | Area under the curve of the local mean field potential computed on ROIs | No |
| AUC GMFP | Area under the curve of the global mean field potential | No |

**Supplementary Table 1:** Summary of features used in the classification of AD vs HC using TMS-EEG data

Supplementary Figure 2 provides an overview of the results of the classification using Random Forest on features computed only after the TMS pulse and features normalized to their baseline value. The performance is higher for all metrics in the case of using regular features computed at a global level. For the ROI based features, the classification performance is slightly improved when features are normalized to baseline.


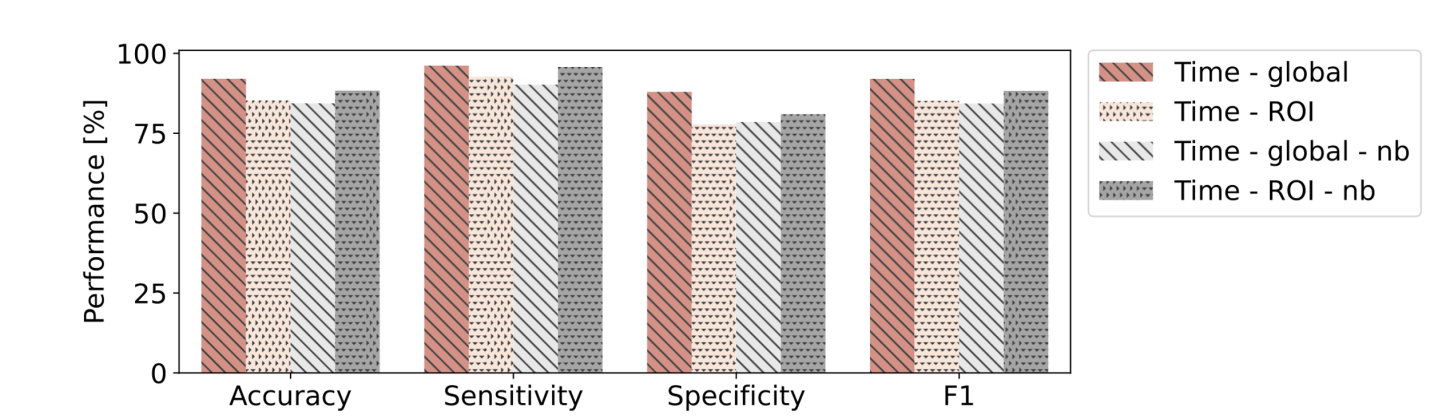


**Supplementary Figure 2:** Performance comparison between results obtained using features normalized to baseline (nb) and features computed only after the TMS pulse.

## 3 Comparison of resting state EEG from baseline recordings and TEP information as input for AD identification

Time domain resting state EEG data has previously been used in the problem of classifying Alzheimer’s disease patients from healthy controls. Our classification results using TMS-EEG data could be validated in comparison to using resting state EEG as input.

For the dataset used in this study, not all participants that underwent the TMS-EEG protocol had also resting state EEG data recorded. For a fairer comparison, we have extracted information from the baseline period of the recordings obtained during the TMS-EEG protocol. A period between 500ms and 200ms prior to the TMS pulse being applied was considered as baseline and assumed equivalent in content to resting state EEG.

A similar processing chain was applied to the baseline data as for the data after the TMS pulse. The segments are divided into epochs based on the timings of the TMS pulse. The resulting epochs for each trial are averaged and descriptive statistical features are extracted. Features that were used to characterize the TEP could not be obtained from resting state data. The extracted information included: maximum and minimum amplitude, skewness, kurtosis, energy, Hjorth activity, mobility, and complexity. As in the case of TMS-EEG classification, features extracted from the baseline are used as input in a RF classifier. The obtained model is validated in a leave-one-subject-out cross-validation.

Classification performance for the models trained on both types of input data can be visualized in Supplementary Figure 3. For both experiments, the extracted features were averaged either globally or per ROI. For the baseline classification, the accuracy, sensitivity, specificity and F1 score was of 93.19%, 96.89%, 89.5% and 93.18% respectively for globally averaged features, while for ROI averaged features, 95.42%, 97.5%, 93.34% and 95.41% respectively.

The performance obtained in our experiments when using TEP information for classification of AD patients is comparable to the classification using baseline information. This shows that TMS-EEG can be an effective means of identifying AD patients from HC.


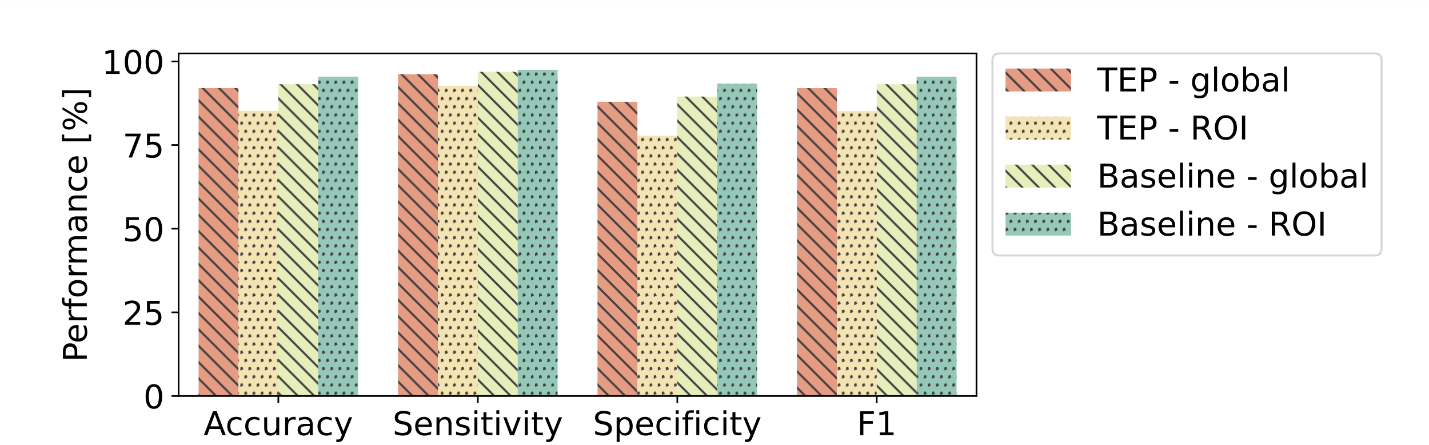


**Supplementary Figure 3**: Comparison of classification performance for the Random Forest classifier trained on information extracted from the baseline EEG and on information extracted from the TEP.
